# Investigating Drivers Impacting Carbon Stock and Carbon Offset in a Large-Scale Rubber Plantation in the Middle South of Thailand

**DOI:** 10.21315/tlsr2024.35.1.8

**Published:** 2024-03-30

**Authors:** Rawee Chiarawipa, Buncha Somboonsuke, Sirima Wandao, Apichet Thongsong, Supet Jirakajohnkool

**Affiliations:** 1 Agricultural Innovation and Management Division, Faculty of Natural Resources, Prince of Songkla University, Hat Yai District, Songkhla 90110 Thailand; 2 Disaster Prevention and Mitigation Regional Center 11, Department of Disaster Prevention and Mitigation, Suratthani, Thailand; 3 Land Development Regional office 12, Land Development Department, Songkhla, Thailand; 4 Department of Sustainable Technology Development, Faculty of Science and Technology, Thammasat University, Pathum Thani, Thailand

**Keywords:** Greenhouse Gas Emissions, Low Carbon Agriculture, Carbon Credit, Voluntary Market, Rubber Agroecology

## Abstract

A large-scale rubber plantation in Southern Thailand is expected to capture a significant amount of carbon dioxide from emissions through carbon sinks in the vegetation and soil. The goal of this research is to create a carbon offset assessment for rubber plantations lasting for 30 years using a voluntary market contract approach. To evaluate the area of large-scale rubber plantations, this study evaluated major growing regions in five provinces in the middle-south region of Thailand (Nakhon Si Thammarat, Phatthalung, Songkhla, Satun and Trang) using an integrated RS-GIS technique that incorporated biomass allometric equations, soil series databases, and object-based classification. The classification of rubber plantation areas and the mapping of rubber stand ages were conducted to estimate the above-ground biomass of the rubber tree. Texture analysis was used in the rubber classification process, and normalised difference vegetation index (NDVI) was combined with texture analysis to separate vegetation areas from other land cover. Four groups of varying ages (1–6, 7–13, 14–20 and 21–30 years old) were evaluated for their capacity to generate carbon offsets. The equations of voluntary market contract revenue according to the contract method of the CCX were applied for this case study. This evaluation was used to estimate their annual value, total and net incomes in the carbon market price regarding the RGGI Allowance (RGA). Carbon offset income was then used to estimate the potential income (over a 30-year period) of the life of the contract. The results showed that the carbon stock potential of rubber plantations depended on the age of the trees and the soil carbon stock. The total carbon stock in the rubber plantations varied from 249.73 to 301.48 Mg C/ha (or equivalently 916.49 to 1,106.44 Mg CO_2_e/ha). Furthermore, the potential net income of the contract was estimated to be between USD5,378.32 and USD5,930.38 Mg CO_2_e/ha over a 30-year period according to the voluntary market contract revenue. These results suggest that the large agricultural land plot policy could create opportunities for carbon offsetting. The policy of large-scale rubber areas could be used as a tool and mechanism for farmers who are considering participating in carbon-crediting mechanisms. Then, farmers could use voluntary market contracts as a guide and foundation for their decision-making. The carbon offset credit strategy could assist Thailand in achieving its climate goals of transitioning to a low-carbon agriculture sector.

**Highlights**

- The combination of biomass models, soil series database, and geoinformatics technology has the potential to facilitate the estimation of carbon stocks in large-scale rubber regions.
- The proportion was in the vegetative parts of rubber trees, litter production, and soil layers, accounting for 69.35%, 1.77%, and 28.88% of overall the total carbon stock in rubber plantations.
- Farmers could earn potential net income of the contract between USD5,378.32 and USD5,930.38 Mg CO_2_e/ha over the entire contract period of 30 years according to the voluntary market contract revenue.

## INTRODUCTION

Southeast Asian countries mainly supply the world’s natural rubber production, and rubber (*Hevea brasiliensis*) is an economically important tree crop in the region (Association of Natural Rubber Producing Countries [ANRPC] 2021). Globally, Thailand is the biggest producer and is the second in the rubber planting area. In 2020, Thailand had a total rubber planting area of 3.96 million (M) ha, in which the southern part of the country occupied a major planting area of 2.30 M ha, providing primary income for approximately six million smallholders (Office of Agricultural Economics 2020).

In addition, rubber planting areas play an important role in carbon dioxide absorption and carbon storage in both above- and below-ground biomass and soil. The rubber tree has high biomass increasing with the age of the tree, and varies in biomass stocks in different vegetative parts of the tree (Hytönen *et al*. 2018). Trees effectively store carbon in the form of plant biomass. The regulatory mechanisms of the plant absorb carbon dioxide through the photosynthesis process, which leads to almost automatically reducing the amount of carbon dioxide amount in the atmosphere (Redondo-Brenes & Montagnini 2006; Holland *et al*. 2019).

Recently, geoinformatics technology that can quickly identify the land cover of trees from satellite images has been used extensively for assessing carbon stock in large geospatial forests (Kafy *et al*. 2023), terrestrial urban environment (Wang *et al*. 2022), and plantations (Goetz & Dubayah 2011; Jeyanny *et al*. 2011). Nowadays, selecting remotely sensed data for land-cover mapping at a regional scale tends to apply either image segmentation or modelling for object identification (Lu *et al*. 2016). Mapping of rubber plantations by the object-based classification provides an opportunity to trace the stages of the plantation and their stand ages (Chen *et al*. 2018).

The integrated use of geographic information systems (GIS), global positioning systems (GPS), and remote sensing (RS) is an effective modern tool for geospatial mapping (Thakur *et al*. 2017). Hence, geospatial technologies, field measurements and allometric equations were combined to enable rapid assessment of vegetative biomass and carbon stock of large areas at high accuracy in a time- and cost-efficient manner (Chen *et al*. 2016). Chen *et al*. (2018) estimated carbon stock in rubber growing areas by combining GIS with a high spatial resolution in Landsat imagery, demonstrating successes in several rubber tree mappings. Moreover, the model was able to estimate the amount of aboveground biomass of rubber plantations using satellite imaging technology (Yasen & Koedsin 2015). The results of this method correspond to the biomass values derived from on-field data measurements such as trunk diameter, height, and age of trees. (Avtar *et al*. 2014). The classification accuracy of rubber tree ages varied widely around 78%–96% as a result of satellite image classification for <7, 7–15 and >15 years (Li & Fox 2012; Suwanwerakamtorn *et al*. 2012; Koedsin & Huete 2015). The maximum carbon stock in a metric ton (Mg) of a 25-year-old rubber plantation was estimated to be 9.14 Mg C/ha/yr (Charoenjit *et al*. 2015).

Raising consciousness concerning climate change and global warming has highlighted the emissions of CO_2_ and other greenhouse gases (GHGs) as potential sources of the phenomenon (Poolen & Ryszka 2021). In order to reach climate neutrality by 2050 and reduce greenhouse gas emissions by at least 55% by 2030, the Paris Agreement outlines a global plan to avert the dangerous effects of climate change and limit global warming to below 2°C, with efforts to keep it below 1.5°C. Additionally, this framework strengthens countries’ ability to respond to the impacts of climate change and encourages their efforts (United Nations Framework Convention on Climate Change 2022). According to the Kyoto Protocol and Paris Agreement on global climate change policies and practices, carbon offset/credit strategies have become a priority among the signatory countries (Intergovernmental Panel on Climate Change [IPCC] 2007; 2019), implemented through either a mandatory or compliance approach, or a voluntary approach (Tarnoczi 2017). Many developed countries, such as the United States of America, Australia and European countries, have been focusing on agricultural carbon markets to reduce GHG emissions and achieve the long-term objectives outlined in the Paris Agreement (Anderson *et al*. 2015).

Since 2015, the adoption of the Paris Agreement has stabilised the activity of carbon credit trading and resulted in significant growth in the Voluntary Emissions Reduction (VER) market (World Bank 2020). The largest markets include the European Union Allowance (EUA), California Carbon Allowance (CCA), RGGI Allowance (RGA), California’s Cap-and-Trade Program, and New Zealand Emissions Trading Scheme (NZ ETS), along with bilateral trading between buyers and development projects (Over-the-Counter: OTC) in both the voluntary and mandatory markets (The EU Emissions Trading System 2017). However, the costs of carbon credits vary depending on the type of credit (Hein *et al*. 2017). Other voluntary exchanges include the newly launched Climate Impact X (CIX) in Singapore, the Carbon Trade Exchange in London and Sydney, the Air Carbon Exchange (ACX) in Singapore, and the Saudi Stock Exchange (Tadawul) (Bose *et al*. 2021).

The findings of the study have been reported that the biomass carbon stock of rubber plantations of above 20-year-old stands ranges from 66 Mg to 170 Mg C/ha in different regions of the world. For example, in Thailand (Petsri *et al*. 2013), China (Yang *et al*. 2005), Ghana (Wauters *et al*. 2008), Brazil (Maggiotto *et al*. 2014), and India (Nath *et al*. 2019).

For decades, the increase in rubber plantation areas has had socioeconomic benefits, such as an increase in per capita income and expenditure, as well as overall smallholding rubber income (Somboonsuke *et al*. 2009). In 2015, Thailand signed the Paris Agreement, committing to reducing emissions by 2030. The large agricultural land plot policy is an innovative approach to agricultural reform for Thai farmers that uses the carbon stock potential of rubber plantation systems as a concept of low-carbon agriculture. Therefore, rubber plantations are a permanent sink for atmospheric CO_2_ and other greenhouse gases (GHGs), which have the potential to mitigate climate change and help achieve the long-term objectives outlined in the Paris Agreement.

To develop a carbon credit income offset model that can be used to manage rubber plantations and reduce greenhouse gas emissions. This study is expected to directly drive low-carbon agricultural mechanisms by supporting farmers and communities to adopt sustainable rubber agroecosystems that reduce greenhouse gas emissions. This will lead to a low-carbon agricultural society. The pattern of carbon offset can be used to implement the carbon credit trading mechanism for rubber plantations in the country in the VER market system. It will also create alternative income for farmers from the carbon credit mechanism. Government agencies, the private sector, and those interested will be able to use this study for policy considerations to concretely drive the low-carbon agricultural sector.

Therefore, this study aimed to assess the geospatial carbon stock of large-scale rubber plantations over 30 years by using biomass allometric equations in combination with a soil series database and geoinformatics technology. To estimate the annual value and incomes in US dollars, the evaluation of voluntary market contract revenue was conducted using the RGGI Allowance (RGA) market as a case study.

## MATERIALS AND METHODS

### Experimental Site Description

**Figure 1:**
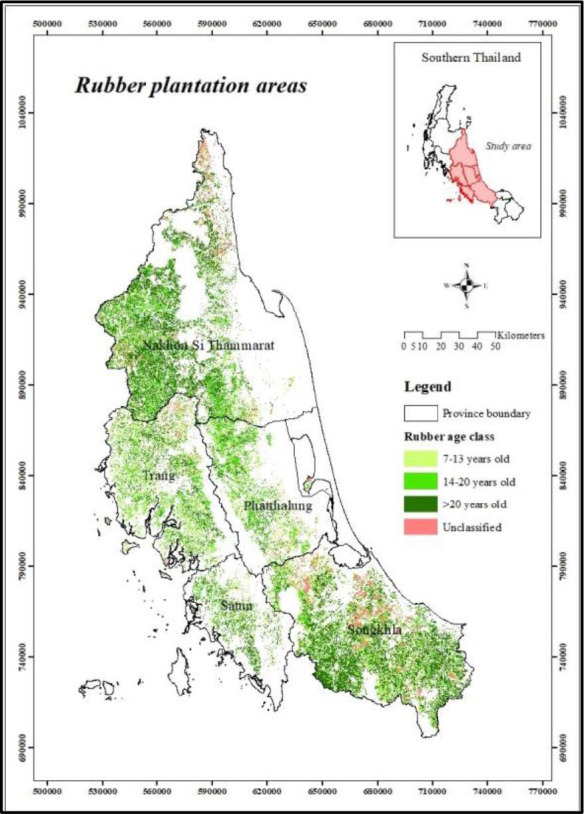
Classification in different ages of 7–13, 14–20 and 21–30 year-old rubber plantations at 5-growing regions of rubber plantations in the middle south of Thailand (Nakhon Si Thammarat [NS], Phatthalung [PT], Songkhla [SK], Satun [ST], and Trang [TR]).

The study focused on the major rubber-growing areas in five provinces of Southern Thailand, namely Nakhon Si Thammarat (NS), Phatthalung (PT), Songkhla (SK), Satun (ST) and Trang (TR), which account for approximately 70% of the rubber-growing area and have the potential for carbon stock and offsetting (Fig. 1) (Office of Agricultural Economics 2020). The study’s reflection of the rubber environment and social factors is quite thorough, and it has some academic significance.

### Geospatial Methods for Rubber Age and Biomass Classification

The biomass of rubber trees of different ages was assessed through measurements taken on plots located in five provinces. An integrated RS-GIS technique was used to map the distribution of rubber tree growth and classify it to cover the entire study area. We applied an integrated RS-GIS technique to map the distribution of rubber tree growth in the area.

We used Landsat 8 Operational Land Imager (OLI) imagery to identify land-use and land-cover types which included urban, village, river basin, forest, agricultural and other areas, approved by the Southern Regional Geo-Informatics and Space Technology Center of Prince of Songkla University. The images were acquired from the Landsat 8 OLI sensor at a resolution of 30 m, and captured at three-year intervals between 2013 to 2015 without cloud cover. The satellite images were converted to Top of Atmosphere (TOA) reflectance and then mosaicked into a seamless single image as described by Sayavong *et al*. (2019). Also, we used high-resolution THEOS (PAN-Sharpened) images with a resolution of 0.5 m to classify the ages of rubber trees and to create a database of themes and layers as described by Yongsatisak *et al*. (2011). In this study, the true colour composite image is created by assigning the visible light bands red (B04), green (B03) and blue (B02) to the corresponding red, green and blue channels. Pixel-based supervised image classification was implemented on multispectral satellite images to classify rubber plantations and other land cover types. Pixel-based classification was employed to categorise numerical data, and NDVI combined with texture analysis was used to differentiate vegetation areas from other land covers. The producer’s and user’s accuracies of rubber stand ages for four groups (< 6, 7–13, 14–20 and 21–30 years old) ranged from 85% to 100%, with overall accuracies of 80% to 100% and a Kappa coefficient of 0.91. Moreover, texture analysis was used for rubber classification. The rubber growing areas were divided into four categories based on the NDVI image texture analysis: (1) 7–13 years old rubber farms; (2) 14–20 years old rubber farms; (3) 21–30 years old rubber farms; and (4) unclassified areas consisting of bare ground, mixed scrub and other crop areas.

### Assessment of Biomass and Carbon Stock in Plant Parts

The amount of biomass in rubber farms was estimated using the empirically allometric biomass equation Redondo-Brenes and Montagnini (2006) as follows:


ln (Y)=a+b ln (DBH)

where ln = natural logarithm, Y = dry weight of rubber tree biomass (Mg/ha), and DBH = diameter at breast height of rubber tree (cm).

The primary data sources from Songkhla province were used to estimate rubber biomass and assess the above- and below-ground carbon stock in the middle south region according to the agro-climatic conditions. Then, above- and below-ground biomass (ABGB) in different vegetative parts of rubber farms were calculated using the following equations (Chiarawipa *et al*. 2012).


$$\begin{array}{l} \text{Leaf: ln ln }({\text{Y}}_{\text{leaf}})=0.8796\;(\text{ln DBH})-0.6974\\ \text{Branch: ln }({\text{Y}}_{\text{branch}})=2.7559(\text{ln DBH})-3.7155\\ \text{Stem: ln }({\text{Y}}_{\text{stem}})=2.2494(\text{ln DBH})-1.8338\\ \text{Root: ln }({\text{Y}}_{\text{root}})=1.9903(\text{ln DBH})-2.8766\\ \text{Litter }(\text{leaf})\text{: L}(\text{leaf})=-0.0179{\text{x}}^{2}+0.6343\text{x}+1.0818\\ \text{Litter }(\text{branch})\text{: L}(\text{branch})=-0.0005{\text{x}}^{2}+0.0203\text{x}+0.0919 \end{array}$$

where L = dry weight of rubber litter biomass (Mg/ha), and x = age of rubber tree (yr).

The biomass of rubber trees of different ages was then evaluated from 1 to 30 years old. The carbon stock in the above-below ground biomass of rubber plantations was then calculated by converting the biomass values.

### Assessment of Soil Carbon Stock in Rubber Plantation

The soil organic carbon (SOC) was estimated by measuring the organic carbon content and bulk density. The soil organic classification was divided into four categories: very low (≤ 0.5%), moderate (> 0.5%–1.5%), high (> 1.5%–2.5%), and very high (> 2.5%) as described by Kungpisdan (2006). At a soil depth of 0 cm–50 cm, 25 soil sets were selected from the Land Development Department’s soil database based on the amount of carbon deposition or sequestration in the rubber plantation area. There are five soil series (no. 17, 18, 40, 43 and 59) with carbon stock lower than 0.50%, 13 soil series (no. 3, 5, 6, 10, 16, 23, 25, 26, 34, 39, 42, 50 and 60) with moderate carbon stock (0.50%–1.50%), five soil series (no. 7, 14, 45, 51 and 53) with high carbon stock (1.50%–2.50%), and two soil series (no. 13 and 32) with very high carbon stock (more than 2.50%).

### Assessment of Carbon Stock in Rubber Plantation

The quantity of above- and below-ground carbon stocks in the rubber farms, including the different parts of rubber trees, litter, and trees, were calculated using the following equation (Zheng *et al*. 2008):


$${\text{CS}}_{\text{t}}=\sum_{\text{i}=1}^{\text{n}}\left({\text{C}}_{\text{Ti}}{\text{B}}_{\text{Ti}}\right)+\sum_{\text{j}=1}^{\text{n}}\left({\text{C}}_{\text{Fj}}{\text{B}}_{\text{Fj}}\right)+\sum_{\text{l}=1}^{\text{n}}\left({\text{C}}_{\text{Sl}}{\text{BD}}_{\text{l}}\right)$$

where CS_t_ = carbon stock in rubber plantation (Mg C/ha), C_Ti_ = the carbon mass proportion in the biomass of rubber tree (45%), B_Ti_ = biomass in rubber parts (Mg C/ha), C_Fj_ = the carbon mass proportion in the litter decomposition (45%), B_Fj_ = biomass in the litter (Mg C/ha), C_Sl_ = soil organic carbon content at 0–50 cm soil depth (%), and BD_l_ = soil bulk density at 0–50 cm soil depth (g/cm^3^). In addition, the geospatial information on the classification of above- and below-ground carbon in the rubber plantations were classified. The carbon sequestration rate (Mg C/ha/yr) in rubber plantations was evaluated in combination with vegetative parts, as well as in litter production, and top and subsoils. Then, carbon sequestration rate in different-aged rubber trees was evaluated in 1 to 30 years old:


$${\text{CS}}_{\text{r}}=\left(\frac{\Delta \text{S}}{\Delta \text{t}}\right)=\frac{\left(\Delta_{{\text{S}}_{\text{f}}-{\text{S}}_{\text{0}}}\right)}{\left(\Delta_{{\text{t}}_{\text{f}}-{\text{t}}_{0}}\right)}$$

where CS_r_ = carbon sequestration rate in rubber plantations (Mg C/ha/year), s_0_, s_f_ = initial and final rates of carbon stock (Mg C/ha), and t_0_, t_f_ = initial and final ages of rubber tree (year).

### Assessment of Carbon Offset in The Rubber Plantation

The carbon sequestration rate (Mg CO_2_e/ha/yr) of rubber plantations in different age stages was categorised into four groups by year: 1–6, 7–13, 14–20 and 21–30 years old. A case study was conducted to estimate the annual value and total income in US dollars under the Regional Greenhouse Gas Initiative (RGGI) Allowance (RGA) market price (RGGI 2023).

The equations of voluntary market contract revenue from the Chicago Climate Exchange (CCX) contract method (Ignosh *et al*. 2009; Current *et al*. 2010) were adjusted to evaluate the income from the carbon offset in the rubber plantation, as defined by Chiarawipa *et al*. (2012), as follows:


$${\text{T}}_{\text{c}}={\text{N}}_{\text{c}}+{\text{R}}_{\text{c}}$$

where; T_c_ = the total income over the duration of the contract (USD), N_c_ = the income from the carbon offset contract (USD), and R_c_ = the income from the carbon reserve pool credit (USD).


$$\begin{array}{l} {\text{N}}_{\text{c}}=({\text{I}}_{\text{a}})-({\text{F}}_{\text{i}})\\ {\text{I}}_{\text{a}}=({\text{C}}_{\text{t}})\times (\text{CTM}) \end{array}$$

where I_a_ = the annual revenue from carbon offset trading (USD/yr), C_t_ = the quantity of carbon offsets traded is 80% of the carbon stock (Mg) in the rubber plantation, CTM = the average trading rate of carbon in trading markets (USD/Mg C), and F_i_ = the fees for the contract (USD).


$${\text{F}}_{\text{i}}=({\text{F}}_{\text{i},\text{a}}+{\text{F}}_{\text{i},\text{v}}+{\text{F}}_{\text{i},\text{c}})$$

where F_i, a_ = fees for aggregators (10%I_a_) (USD), F_i, v_ = project verification fees (USD0.15/Mg C) (USD), and F_i, c_ = carbon trading fees (USD0.20/Mg C) (USD).


$${\text{R}}_{\text{c}}=({\text{I}}_{\text{r}})-({\text{F}}_{\text{j}})$$

where I_r_ = carbon offsets from a carbon reserve pool (USD), and F_j_ = the fees for verifying the project (USD).


$${\text{I}}_{\text{r}}=({\text{C}}_{\text{r}})\times (\text{CTM})$$

where C_r_ = carbon reserve pool (20%C_s_) (Mg), and CTM = the average trading rate of carbon in trading markets (USD/Mg C),


$${\text{F}}_{\text{j}}=({\text{F}}_{\text{j},\text{a}}+{\text{F}}_{\text{j},\text{v}}+{\text{F}}_{\text{j},\text{c}})$$

where F_j, a_ = contract fees with an aggregator (10%I_r_) (USD), F_j,v_ = project verification fees (USD0.15/Mg of C_r_) (USD), and F_j, c_ = fees for carbon market trading (USD0.20/Mg of C_r_) (USD).

Also, the sequestration rates of carbon dioxide equivalent (CO_2_e) was calculated by a conversion factor of carbon dioxide absorption (3.67) (McPherson 1998). Then, voluntary market contract revenue was used to estimate the potential income from the carbon sequestration credit of the 30-year life span of rubber plantations in the five rubber growing regions in the middle south of Thailand.

## RESULTS

### Carbon Stock in Rubber Parts and Litter Production

**Figure 2:**
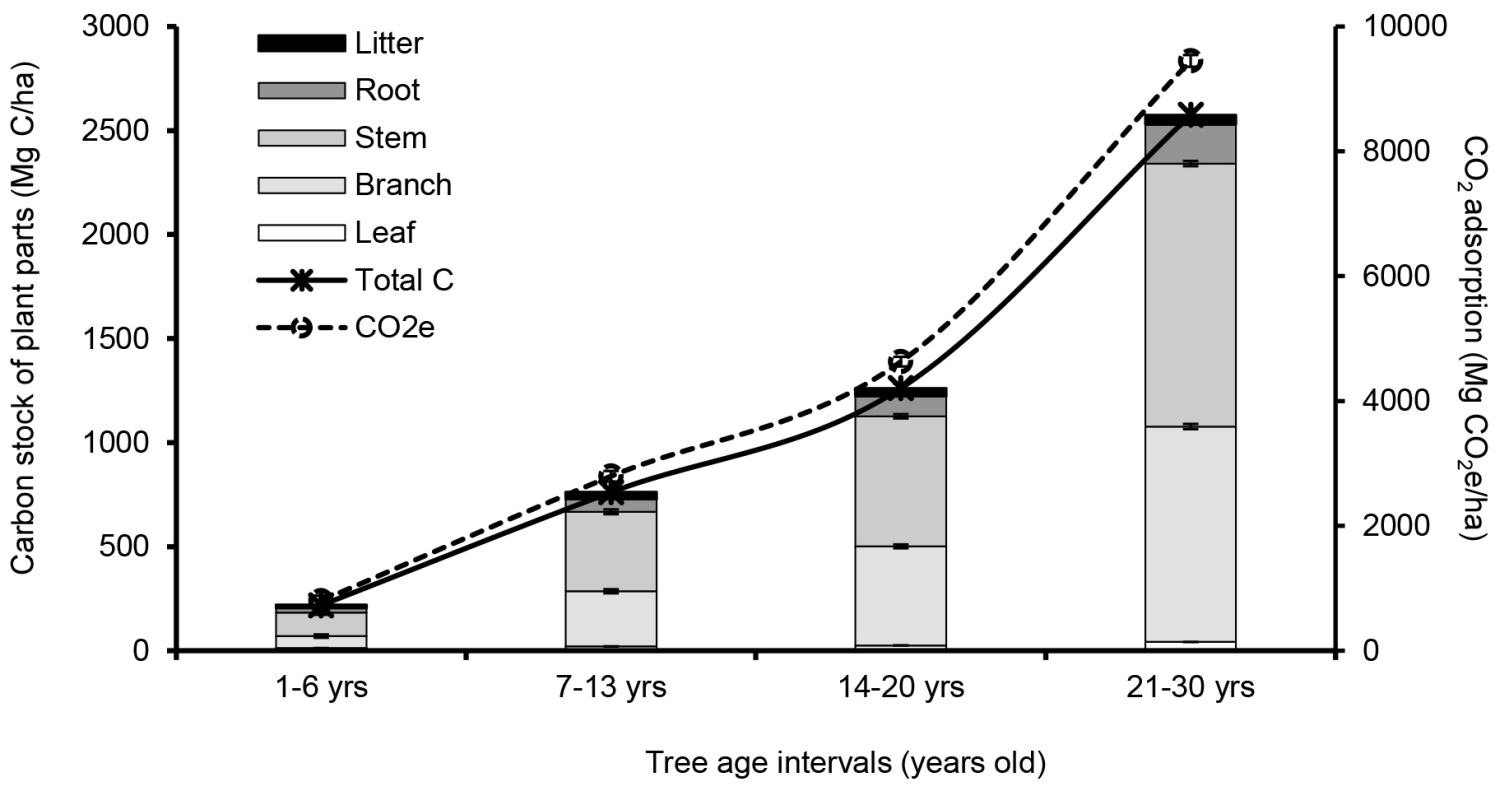
Carbon stock assessment of the interval accumulations in rubber tree and litter production.

Carbon stock in the vegetative parts of rubber trees increased with age levels (Fig. 2). Rubber trees had the highest carbon stock between 21 to 30 years of age. The highest accumulations in the stem were 1,263.62 Mg C/ha (or 126.36 Mg C/ha/yr), followed by the branches, roots and leaves with 1,035.81, 188.52 and 41.73 Mg C/ha (or 103.58, 18.85, and 4.17 Mg C/ha/yr), respectively. Carbon stock in rubber trees had tended to increase by year, especially in the trunk section with the highest carbon storage capacity.

The rubber trees in the age range of 21 to 30 years old could store the highest carbon stock of 2,529.68 Mg C/ha (or 252.97 Mg C/ha/yr) in all parts, followed by the 14–20, 7–13 and 1–6 years old groups, which had the carbon stocks of 1,223.93, 729.80 and 205.00 Mg C/ha (or 174.85, 104.26 and 34.17 Mg C/ha/yr), respectively.

It was found that most carbon stock in litter production was accumulated in the leaf part over 95%. The age range between 14–20 and 21–30 years old rubber plantation was the highest carbon stock in little production at 37.83 and 44.96 Mg C/ha (or 5.40 and 4.50 Mg C/ha/yr), followed by 1–6 and 7–13 years old rubber plantations, which had the carbon stock values of 14.95 and 31.78 Mg C/ha (or 2.49 and 4.54 Mg C/ha/yr), respectively. In addition, the rubber trees had a high potential to capture further carbon dioxide by adsorption processing, approximately 800.65, 2,794.99, 4,630.64 and 9,448.93 Mg CO_2_e/ha (or 133.44, 399.28, 661.52 and 944.89 Mg CO_2_e/ha/yr), respectively.

### Carbon Stock in Soil

This study found that the soil series under the rubber plantations had moderate and higher carbon stock. There is a tendency for highly soil fertility. All of these soil groups had organic carbon (OC) values in the range of 0.75% to 3.25% and organic matter (OM) in the range of 1.10% to 5.60%.

**Figure 3:**
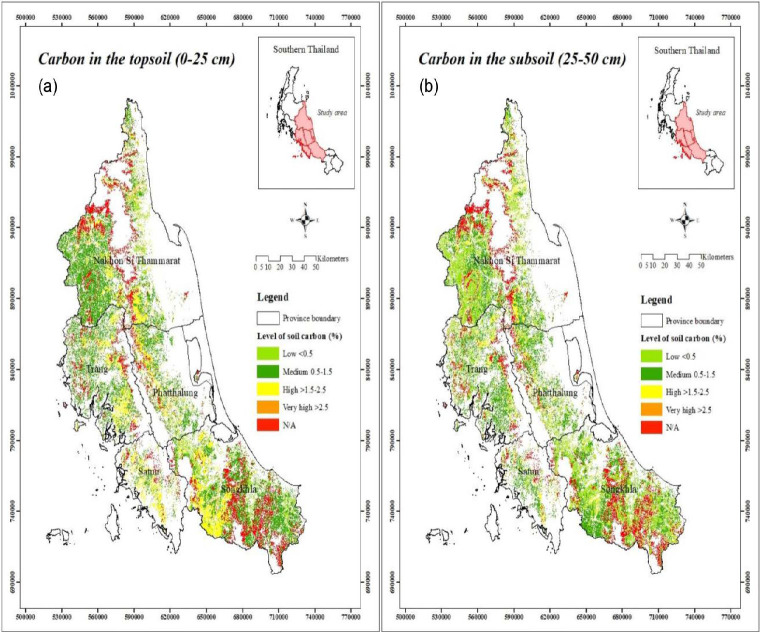
Classification of soil carbon stock at the topsoil (0–25 cm) (a) and subsoil (25–50 cm) (b) in 5-growing regions of rubber plantations in the middle south of Thailand (Nakhon Si Thammarat (NS), Phatthalung (PT), Songkhla (SK), Satun (ST) and Trang (TR)), N/A = Not available soil series data.

The rubber plantations in Satun province had the highest soil carbon accumulation in the topsoil layer (0 cm–25 cm depth) at 55.66 Mg C/ha, followed by that of Phatthalung, Trang, Nakhon Si Thammarat, and Songkhla provinces at 45.82, 41.23, 38.15 and 36.41 Mg C/ha (Fig. 3a), respectively.

In the subsoil at a depth of 25 cm–50 cm (Fig. 3b), Satun province had the highest soil carbon stock value which was 45.42 Mg C/ha, followed by Phatthalung, Trang, Nakhon Si Thammarat, and Songkhla provinces, with the storage value of 37.46, 32.99, 30.14 and 27.54 Mg C/ha, respectively.

### Geospatial Carbon Stock Potential in Rubber Plantations

**Figure 4:**
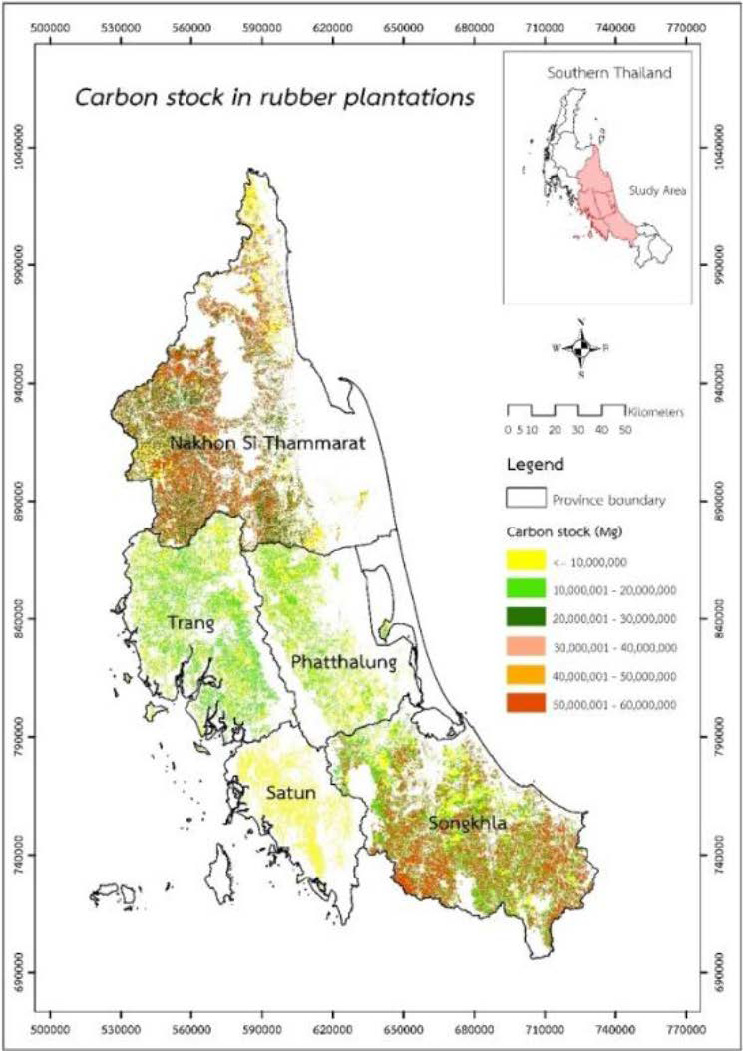
A GIS-based approach for quantifying mapping of carbon stock (Mg) in 5-growing regions of rubber plantations in the middle south of Thailand (Nakhon Si Thammarat (NS), Phatthalung (PT), Songkhla (SK), Satun (ST) and Trang (TR)), N/A = Not available soil series data.

**Figure 5:**
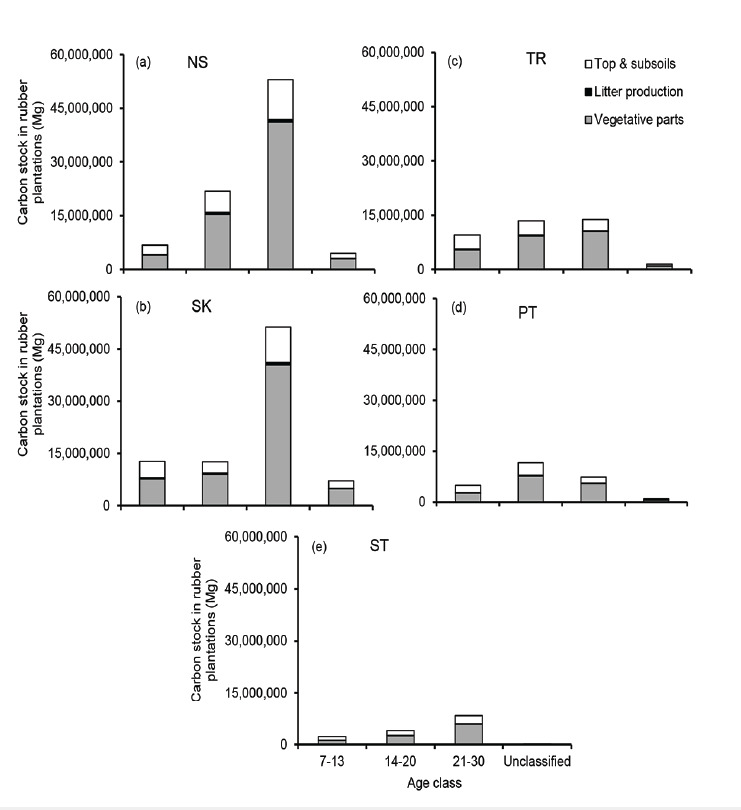
The age classification of carbon stocks in vegetive parts, litter production, and top-subsoils in rubber plantations in Nakhon Si Thammarat (NS)(a), Songkhla (SK) (b), Trang (TR) (c), Phatthalung (PT) (d), and Satun (ST) (e) provinces

The estimation of carbon stock potential in mature rubber plantations (7–30 years old aged) was shown on the geospatial mapping in Fig. 4. The quantification of total carbon stock in 5-growing regions of rubber plantations in the middle south of Thailand is presented in Fig. 5.

**Figure 6:**
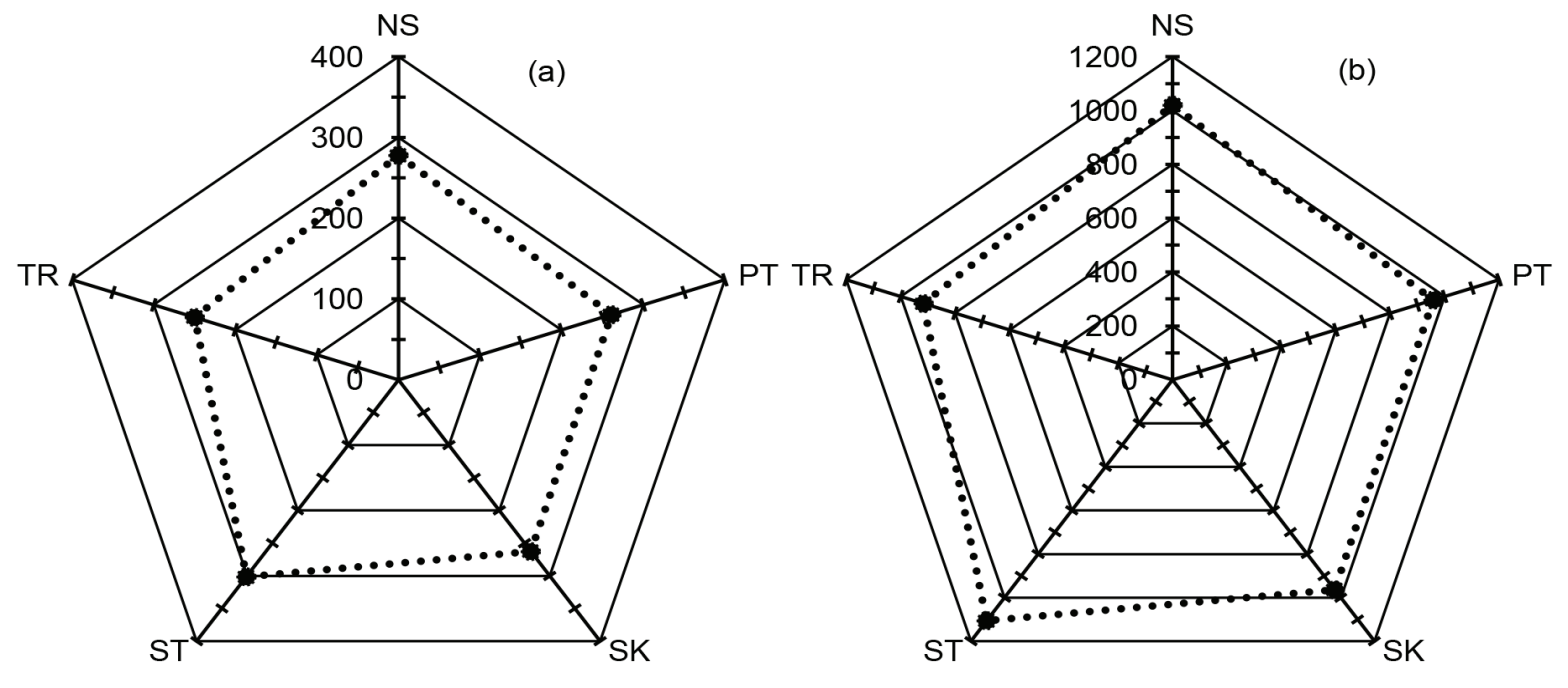
The geospatial distribution of total carbon stock in Mg C/ha (a) and in Mg CO_2_e/ha (b) in 5-growing regions of rubber plantations in the middle south of Thailand (Nakhon Si Thammarat (NS), Phatthalung (PT), Songkhla (SK), Satun (ST) and Trang (TR)).

The rubber plantations in Nakhon Si Thammarat (NS) province (Fig. 5a), having a rubber plantation area of 309,671.77 ha, had the highest carbon storage of 86.04 M Mg C (277.85 Mg C/ha), followed by Songkhla (SK) (Fig. 5b), Trang (TR) (Fig. 5c), and Phatthalung (PT) (Fig. 5d), owning 319,423.80, 152,814.88, and 95,624.54 ha, stored 83.86 (262.56 Mg C/ha), 38.16 (249.73 Mg C/ha), 24.97 (261.14 Mg C/ha) M Mg C, respectively. The province with the least amount of carbon stock in rubber plantations was Satun (ST) province (Fig. 5e). It had the minimum rubber plantation area of 48,925.44 ha and could store all carbon of around 14.75 M Mg C (301.48 Mg C/ha). Moreover, our results indicate that the carbon stocks in vegetative parts, litter production, and soil layers in the study area ranged from 170.68 to 204.82, 4.63 to 4.91, and 68.29 to 101.08 Mg C, respectively. Then, the geospatial distribution of total carbon stock in Mg C/ha and Mg CO_2_e/ha is presented in Fig. 6.

### Carbon Credit Assessment in Rubber Plantations

The annual sequestration rate of carbon credits measured in Mg of CO_2_e covering all the five provinces ranged between USD191.20 and USD268.87 Mg CO_2_e/ha/yr (Table 1). The lowest offset was found in Songkhla province at USD6,492.22 Mg CO_2_e/ha for 30 years contract. Meanwhile, the rubber plantations in Satun showed the highest income from the carbon offset of USD7,158.62 Mg CO_2_e/ha from carbon credit over the 30-year contract period. In the other provinces, the net income from the carbon offset ranged from USD5,378.32 to USD5,930.38 Mg CO_2_e/ha.

## DISCUSSION

### Above- and Below-Ground of Carbon Stock in Rubber Plantations

Regarding biomass accumulation, the rubber trees could store carbon in different vegetative parts, increasing with the age of the rubber plantation. This study found that the carbon stock in the rubber parts aged 14–20 and 21–30 years was 174.85 to 252.97 Mg C/ha.

Allometric models are widely used in studies of tree biomass and age relationship and are considered to be highly accurate and spatially precise. Furthermore, there is a strong correlation with biomass-age values of up to 98% accuracy as reported by Bing *et al*. (2010). In this study, similar to Hytönen *et al*. (2018), the biomass of all rubber components increased with increasing tree size. However, a study reported that in some provinces in Northeastern Thailand, such as Nong Khai and Khon Kaen, rubber plantations aged 20 years might have a lower biomass and carbon stock of around 3 to 4 times, or only 80.57 and 65.18 Mg C/ha, respectively (Saengruksawong *et al*. 2012; Puttaso 2015). Then, it implied that climate zone factors in the southern region could be probably increase growth and carbon stock potential in the rubber plantation.

At the same time, the soil in the rubber plantation is still one of the carbon reserves from various soil groups in the study area. A high amount of carbon stock was found in the range of 36.41 to 55.66 Mg C/ha in both topsoil (0–25 cm depth) and subsoil (25–50 cm depth) of the studied regions. It indicates that soil sets with high carbon stocks (1.50%–2.50%) and very high carbon stocks (>2.50%) were found to be distributed according to the amount of carbon deposition or sequestration in the rubber plantation area. Consequently, this is likely to be the reason why a high value of SOC was in Satun (ST) province.

The topsoil layer (0–25 cm) in this study was comparable to previous studies which reported that different ages of rubber plantations had high soil carbon stocks of 46.62 Mg C/ha (Chiarawipa *et al*. 2012) and 37.36 Mg C/ha (Saengruksawong *et al*. 2012). Puttaso (2015) observed that soil fertility in rubber plantations aged 3 to 27 years in Khon Kaen province in Northeastern Thailand had the highest soil carbon stock value at 24.59 Mg C/ha.

The results indicate no relationship between soil carbon content and the age range of rubber plantations. It was because the amount of soil organic carbon is directly related to soil organic matter, which varies with agricultural practice and management (Piñeiro *et al*. 2006; Pibumrung *et al*. 2008). For example, rubber plantations at the age of 7, 13, 19, 25 and 47 years with soil carbon stock values were 129.10 to 146.00 Mg C/ha which did not vary with the age of the rubber plantations (Liu *et al*. 2017). Soil organic carbon (SOC) content in rubber plantations was also related to soil physical properties such as bulk density, soil moisture, litter production (N’Dri *et al*. 2018), and annual soil microbial degradation activity (Panklang *et al*. 2022). Because of the ability of rubber trees have been high potential for carbon absorption and accumulation in the plantation soils. Also, SOC is widely used as a more important index of soil quantification and quality that is necessary to evaluate the status of the soil carbon stock in the agricultural area (Zhang & Zhang 2003; Zhang *et al*. 2007). It indicates that the improvement of soil properties with the aging of rubber plantations is one of the reasons why large-scale rubber plantations are important for carbon stock potential. Therefore, this study indicates that soil series is more appropriate to evaluate SOC in a large-scale rubber plantation area to facilitate the non-destructive approach of soil carbon stock geospatial and age specifically.

### Geospatial Carbon Stock Potential in Rubber Plantations

This study could estimate carbon stock in a large-scale rubber plantation region. All plantations had the potential to store carbon of more than 14 to 86 M Mg C in all parts of rubber trees and both topsoil and subsoil layers. This study shows that the age of rubber plantation areas and their carbon stocks could assess using satellite imagery. Rubber plantations across all provinces typically store carbon values between 249.73 and 301.48 Mg C/ha over a period of 1 to 30 years, equivalent to the annual sequestration rate of approximately 10.65 to 11.89 Mg C/ha/yr. Regarding the distribution of carbon stocks, the findings of the object-based classification showed that the highest proportion was in the vegetative parts of rubber trees, litter production, and soil layers (Fig. 5), accounting for 69.35%, 1.77%, and 28.88%, respectively, of overall the total carbon stock in rubber plantations.

The combination of biomass models and object-based classification has facilitated the estimation of carbon stocks in the rubber plantation. This study agrees with the findings of Charoenjit *et al*. (2015), which estimated that all rubber parts in eastern Thailand sequester approximately 130 Mg C/ha over 30 years. Even though there is an 85%–95% consistency between the assessment of rubber stand ages using satellite imagery and on-field classification of rubber biomass (Koedsin & Huete 2015), the performance of biomass estimation using artificial neural networks might be improved to accurately and should be taken into account in future study (Yasen & Koedsin 2015). However, there is an inherent limitation of the data collection process that may have influenced the results. Although this study was able to estimate above-ground carbon stock using an integrated RS-GIS technique, the reflection values of fruit trees intercropped with rubber trees were similar to those of mature rubber trees. As a result, the NDVI and texture analysis values might not be separated.

According to total carbon stock in rubber plantations, in other regions at the age range of 20 to 30 years old could be estimated to be 266.56 Mg C/ha and 111.46 Mg C/ha in the Chachoengsao and Nong Khai provinces (Saengruksawong *et al*. 2012; Puttaso 2015). Meanwhile, the estimated carbon stock in a rubber plantation aged 7 to 47 years old in Southern China was between 159.6 to 291.9 Mg C/ha (Liu *et al*. 2017). However, in a rubber plantation in the Philippines, the carbon stock values for 10 and 20-year-old plantations were 46.79 and 257.95 Mg C/ha, respectively, while that of a 40-year-old plantation was 468.69 Mg C/ha (Corpuz *et al*. 2014). The carbon stock in rubber plantations can vary depending on the environment, so it is important to consider the climate or soil fertility of each region when choosing a planting area (Wauters *et al*. 2008). This is especially important when assessing the impact of biomass carbon stock in rubber trees.

### Carbon Credit Offset Potential in The Rubber Plantation

When looking at the carbon stock potential of large-scale rubber plantations in the mid-Southern region, it was found that farmers could earn net incomes ranging from USD5,378.32 to USD5,930.38 Mg CO_2_e/ha over the entire contract period of at least 30 years. The study results should be used as a guideline and basis for decision-making on participation in the carbon market program for farmers, related agencies, and concerned governments. However, contracts for carbon offset credits in agricultural areas may differ. Carbon offset income may be valued differently depending on the contract method and changing market prices. For example, some organisations may not charge a fee for contract audits (verification fee). Payment schedules may vary between aggregators and projects, and can be semiannual or annual (Ignosh *et al*. 2009; Current *et al*. 2010), or may not include a 20% contract fee for the reserve carbon credit (Farlee & Stelzer 2008). Moreover, the costs may relate to negotiation or benefit sharing with local land users and farmers. Additional income could achieve by selling biodiversity or watershed management credits. But transaction costs can be set between USD1.50 to USD3/Mg CO_2_e on average (Hein *et al*. 2017). This study indicates that potential carbon offset will promote responsible rubber agroecology to access the importance of sustainability in rubber plantations. In addition, some risks associated with primary offsets and procurement approaches should be concerned (Tarnoczi 2017). Then, the estimated carbon sequestration rates and potential carbon offset income from rubber plantations, compared to current market prices, would provide valuable information for decision-making and incentivising participation in carbon-crediting mechanisms.

Currently, voluntary carbon markets with VER credits are becoming increasingly important in the agricultural sector. Since 2009, carbon stock has been able to be traded as carbon credit offsets, such as cultivated maize, soybean (Al-Kaisi *et al*. 2005), rice (Watkins *et al*. 2009) and pasture (Stephenson *et al*. 2004) areas, which are recognised as carbon credits in carbon trading contracts. Therefore, if the carbon credit markets are still growing, consequently, rubber farmers may have more opportunities. Then, the VER price continues to grow with no expected increase in transaction costs, and farmers would be more attracted to participate in the emission offset system. Moreover, endorsing farmers for carbon-crediting mechanisms by emphasising the co-benefits and associated economic incentives should recommend, instead of prioritising its potential financial gains. This indicates that there would be potential to develop rubber plantations in voluntary carbon credit trading projects as an agricultural sector long enough for carbon offset contracts. Then, the assessment of carbon offset could be applied to the carbon offset program in the rubber plantation.

Although the cost-effectiveness may vary by enhancing potential carbon stock in rubber plantations, other factors influencing the management for reducing GHG emissions during pre- and tapping stages need to develop further comprehensive approaches. Moreover, the detailed contractual requirements may cause the farmers’ obligations. For example, the farmer could not be able to change the condition of rubber plantations for at least seven years, and required carbon stock or reducing GHG at or above 16,000 Mg CO_2_e/yr as defined by the contract condition of Thailand Voluntary Emission Reduction Program (T-VER) (The Thailand Greenhouse Gas Management Organization [TGO] 2022).

The government should support a pilot project of carbon credit trading for rubber farmers. It is also necessary to recommend voluntary and social contexts for rubber farmers. An accurate geospatial database about carbon stock in the rubber region and information on rubber plantation management may enhance productivity for driving a low-carbon agriculture sector. Also, the voluntary emission reduction project could help to achieve Thailand’s climate goals for net-zero GHG emissions by 2050.

## CONCLUSION

The geospatial information could apply to assess tree age ranges and carbon stock in rubber plantations efficiently. The highest carbon stock had been found in the rubber plantations aged 21 to 30 years at an average of 257.46 Mg/ha/yr or 944.89 Mg CO_2_e/ha/yr. Also, the soil carbon stock ranged from 36.41 to 55.66 Mg C/ha. The total carbon stock in the rubber plantations ranged from 249.73 to 301.48 Mg C/ha (or equivalently 916.49 to 1,106.44 Mg CO_2_e/ha), over 30 years. The potential income for farmers participating in the compensation contract of carbon credit will have net income in the range from USD5,378.32 to USD5,930.38 Mg CO_2_e/ha during the 30-year-participating period. Moreover, this carbon offset method could apply to the carbon offset program in the rubber plantation. The management for reducing GHG emissions during the pre-and-tapping stages needs to develop further comprehensive approaches.

## Figures and Tables

**Table 1:** Estimated potential income (30-year period) of the life of contract for carbon offset in the 5-rubber growing region in the middle south of Thailand (Nakhon Si Thammarat (NS), Phatthalung (PT), Songkhla (SK), Satun (ST) and Trang (TR)).

Sequestration rate of CO_2_e (yr)^a^	Carbon offset income (USD/Mg CO_2_e/ha/yr)^b^				

	Nakhon Si Thammarat (NS)	Phatthalung (PT)	Songkhla (SK)	Satun (ST)	Trang (TR)
1–6	249.25	258.73	246.66	268.87	252.81
7–13	232.87	242.64	230.27	252.49	236.42
14–20	215.22	224.99	212.63	234.84	218.78
21–30	193.79	203.56	191.20	213.41	197.35

Total income (USD/Mg CO_2_e/ha)	6,570.10	6,861.38	6,492.22	7,158.62	6,676.79

	Fees and deductions of contract revenue^b^				

Carbon reserve pool (20%)	1,314.02	1,372.28	1,298.44	1,431.72	1,335.36
Aggregator fee (10%)	657.01	686.14	649.22	715.86	667.68
Verification fee (USD0.15/Mg CO_2_e)	201.54	210.47	199.15	219.59	204.81
RGA exchange fee (USD0.20/Mg CO_2_e)	268.72	280.63	265.53	292.79	273.08
Net annual contract payment	4,128.82	4,311.86	4,079.87	4,498.66	4,195.86
Net income (USD/Mg CO_2_e/ha)	5,442.84	5,684.14	5,378.32	5,930.38	5,531.22

*Notes*:

a Sequestration rates of carbon dioxide equivalent (CO_2_e) was evaluated from 1 to 30 years old;

b The average carbon market price of RGA used for the case study was USD4.90/Mg CO_2_e from 2009 to 2022.
